# Comparing Regenerative Biologics and Standard Pharmacotherapy for Chronic Rotator Cuff Tendinopathy: A Study of PRP, Cell-Based, and Peptide Interventions

**DOI:** 10.26502/josm.511500247

**Published:** 2026-01-06

**Authors:** Andre Aabedi, Devendra K. Agrawal

**Affiliations:** Department of Translational Research, College of Osteopathic Medicine of the Pacific, Western University of Health Sciences, Pomona, California, USA

**Keywords:** Cell-based therapy, Chronic Rotator Cuff Tendinopathy, Corticosteroids, Inflammation, Mesenchymal Stem Cells (MSCs), Orthobiologics, Peptide-based Therapies, Platelet-rich Plasma (PRP), Regenerative Medicine, Shoulder Pain Management, Tendon Healing, Tissue Engineering

## Abstract

Chronic rotator cuff tendinopathy is a degenerative condition characterized by persistent shoulder pain, weakness, and functional limitation. Conventional pharmacologic therapies—including nonsteroidal anti-inflammatory drugs and corticosteroid injections—are commonly used but provide only short-term symptom relief without addressing underlying tendon degeneration, and may negatively affect tendon integrity. These limitations have prompted increasing interest in regenerative therapies aimed at promoting tissue repair and durable clinical improvement. This literature review evaluates and compares the clinical outcomes, safety profiles, and practical considerations of regenerative therapies—specifically platelet-rich plasma, mesenchymal stem cell–based interventions, and peptide-based therapies—relative to traditional pharmacologic management for chronic rotator cuff tendinopathy. A comprehensive review of randomized controlled trials, systematic reviews, meta-analyses, and consensus guidelines was conducted to assess pain relief, functional outcomes, tendon structural integrity, safety, and clinical applicability of pharmacologic versus regenerative treatment strategies. Pharmacologic treatments, particularly NSAIDs and corticosteroid injections, demonstrate limited efficacy beyond short-term pain reduction and do not promote tendon healing; repeated corticosteroid use is associated with tendon weakening and inferior long-term outcomes. In contrast, PRP consistently provides superior intermediate- and long-term improvements in pain and function compared with corticosteroids, with favorable safety profiles. Mesenchymal stem cell therapies show promising regenerative potential, including structural tendon improvement and sustained symptom relief, though evidence is limited by heterogeneity, cost, and regulatory constraints. Peptide-based therapies represent an emerging modality with encouraging preclinical and early clinical data but remain insufficiently validated for routine use. Regenerative therapies—particularly platelet-rich plasma and mesenchymal stem cell–based interventions—offer safer and more durable alternatives to conventional pharmacologic management for select patients with chronic rotator cuff tendinopathy who have failed conservative care. However, widespread clinical adoption is limited by variability in protocols, regulatory barriers, and a lack of large-scale, high-quality randomized trials. Further research is required to standardize treatment approaches, refine patient selection, and establish long-term efficacy.

## 1. Introduction

Chronic rotator cuff tendinopathy is a prevalent degenerative disorder marked by shoulder pain, reduced strength, and loss of function. It commonly arises from repetitive mechanical stress, age-related tissue deterioration, and impaired tendon repair processes. The underlying pathology features disrupted collagen architecture, neovascularization, and sustained mild inflammation rather than acute inflammatory changes [1–11]. Co-mobidities, including metabolic syndrome with hyperglycemia and hyperlipidemia, exacerbate the pathophysiological changes and the outcome in rotator cuff injury and healing of the tendon repair [12–20]. Mitochondrial biogenesis and biomechanical properties of the tendon are significantly affected leading to chronic tendinopathy [21–23]. Transcriptional and post-translational mechanisms, including epigenetic and miRNA alterations are the key intracellular events [24–28].

Current pharmacological options have significant drawbacks. Non-steroidal anti-inflammatory drugs (NSAIDs) provide limited pain reduction in rotator cuff pathology, with their modest benefits offset by concerns about renal, cardiovascular, and gastrointestinal complications, particularly during prolonged therapy [4,29]. Acetaminophen demonstrates minimal efficacy for musculoskeletal pain or functional improvement, while opioids are discouraged given their risks and lack of demonstrated superiority [4].

Corticosteroid injections may temporarily alleviate pain but fail to produce durable improvements and may compromise tendon integrity. Evidence from meta-analyses and clinical practice guidelines shows corticosteroid effectiveness is confined to brief periods (3–6 weeks), with no meaningful long-term enhancement of pain control or functional capacity [29,30]. Repeat corticosteroid administration is not advised due to potentially harmful effects on tendon structure, including elevated rupture risk and compromised healing capacity, particularly problematic if surgical intervention becomes necessary [4,31,32]. Both NSAIDs and corticosteroids are most appropriately employed as supportive measures to enable therapeutic exercise, which represents the primary treatment modality [2,3,29,32].

**Figure 1:**
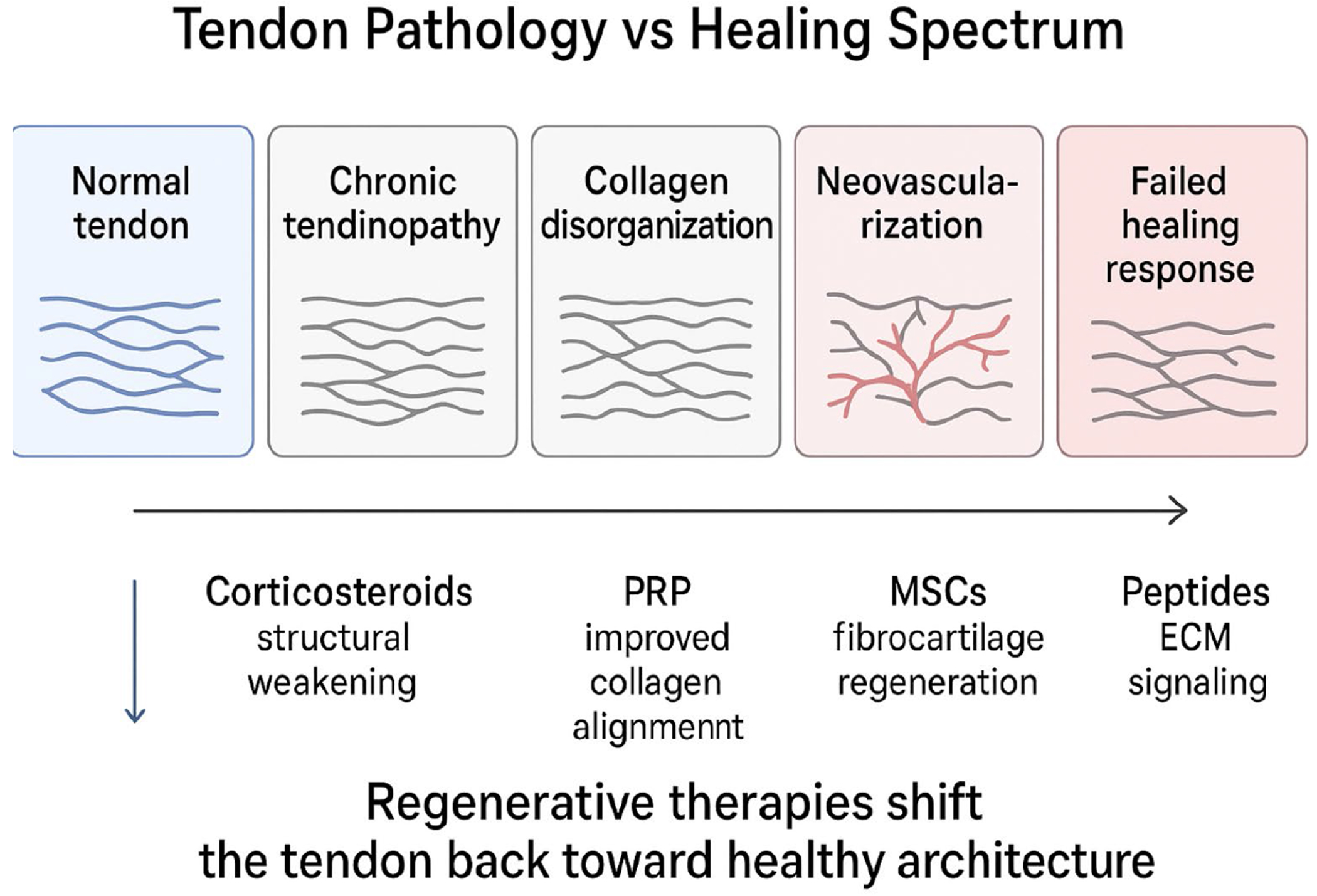
The schematic diagram depicts tendon pathology as a continuum from normal structure to a failed healing response and illustrates how regenerative therapies [platelet-rich plasma (PRP), mesenchymalstem cells (MSCs), and peptides], unlike corticosteroids, aim to restore tendon architecture by improving collagen organization and extracellular matrix signaling rather than providing only symptomatic relief.

In conclusion, pharmaceutical management of chronic rotator cuff tendinopathy faces constraints of limited effectiveness and potential harm, especially with serial corticosteroid use. These shortcomings have sparked growing interest in regenerative approaches, including platelet-rich plasma (PRP), cellular therapies, and peptide-based treatments, which may deliver superior long-term results and enhanced tendon repair [33–41] (Figure 1).

## 2. Pharmacologic Management

Nonsteroidal anti-inflammatory drugs offer limited short-term analgesia in chronic rotator cuff tendinopathy but do not influence tendon repair mechanisms or sustained functional recovery. NSAIDs are frequently prescribed for symptom management in rotator cuff tendinopathy. Systematic reviews and meta-analyses demonstrate that oral NSAIDs achieve temporary pain reduction without improving functional capacity or facilitating tendon restoration. Their pain-relieving properties may enable patient participation in rehabilitation protocols, which constitute essential treatment components, yet NSAIDs fail to target the fundamental degenerative pathology characteristic of tendinopathy. Since chronic tendinopathy involves predominantly degenerative rather than inflammatory processes, anti-inflammatory agents show minimal capacity to alter disease trajectory [1,5–11].

NSAIDs do not augment tendon repair or tissue regeneration. Available evidence does not support NSAID-mediated improvements in tendon architecture or healing capacity, and their administration does not correlate with enhanced long-term clinical results [2]. Moreover, experimental studies indicate that NSAIDs may potentially compromise tendon healing through prostaglandin synthesis inhibition, a pathway implicated in tissue restoration. Consequently, NSAIDs are most appropriately utilized for temporary symptomatic management rather than as interventions capable of modifying the disease process [3].

Safety profiles warrant careful consideration. NSAID use entails gastrointestinal, renal, and cardiovascular risks, particularly with extended treatment duration or in vulnerable patient populations. Topical NSAID formulations may provide analgesia with reduced systemic complications, though robust evidence supporting their application in rotator cuff tendinopathy remains limited [42].

Corticosteroid injections deliver prompt symptom amelioration in chronic rotator cuff tendinopathy but carry risks of tendon deterioration and inferior long-term results relative to regenerative treatment modalities. Corticosteroid injections are extensively utilized for their powerful anti-inflammatory properties and swift pain reduction, generally evident within the initial weeks following administration [43]. Meta-analyses and randomized controlled trials uniformly demonstrate that corticosteroids surpass regenerative approaches such as platelet-rich plasma in early time periods (up to 6 weeks) for alleviating pain and enhancing function [44]. Nevertheless, these improvements are temporary, and corticosteroids fail to deliver sustained benefit beyond the initial treatment phase [45].

Tendon structural deterioration represents a substantial concern with corticosteroid administration 33]. While the available literature does not provide direct quantification of tendon degeneration, editorial analyses and clinical investigations emphasize the potential for corticosteroids to compromise tendon repair processes, elevate rupture risk, and adversely impact surgical outcomes if repair becomes necessary [2,4,16]. This is corroborated by evidence indicating that corticosteroids may exert harmful effects on tendon architecture and regenerative capacity, particularly with repeated administration [30].

Inferior long-term clinical outcomes are well-established. Multiple systematic reviews and meta-analyses reveal that at intermediate and extended timepoints (beyond 3-6 months), corticosteroid injections demonstrate comparable or worse performance than regenerative therapies such as platelet-rich plasma regarding pain control, functional restoration, and rates of subsequent intervention or surgical management [42]. Platelet-rich plasma and alternative regenerative modalities may provide more durable clinical improvements and reduced treatment failure or surgical conversion rates. Additionally, physical therapy as monotherapy or combined with regenerative approaches may represent a more favorable strategy for sustained management, given the absence of lasting corticosteroid benefit and their potential adverse sequelae [46–48].

NSAIDs and corticosteroid injections provide short-term pain relief in chronic rotator cuff tendinopathy but do not promote tendon healing, with corticosteroids carrying risks of tendon deterioration and inferior long-term outcomes compared to regenerative therapies and structured rehabilitation.

## 3. Regenerative Therapies

Regenerative therapies aim to stimulate true tissue repair rather than symptom suppression. Platelet-rich plasma has emerged as a noteworthy regenerative intervention for chronic rotator cuff tendinopathy, demonstrating potential benefits compared to conventional pharmacological approaches, particularly corticosteroid administration [1]. Platelet-rich plasma is an autologous biological preparation concentrated with platelets, growth factors, and cytokines that may facilitate tendon repair and regulate inflammatory responses [34,49]. Numerous systematic reviews and meta-analyses indicate that platelet-rich plasma injections maintain a favorable safety profile and yield substantial improvements in pain severity and shoulder function, particularly at intermediate and extended follow-up intervals, when compared to baseline measurements and corticosteroid injections [47,50,51]. For instance, platelet-rich plasma has demonstrated superior and durable analgesia with enhanced functional outcomes at 6 and 12 months relative to corticosteroids in randomized controlled trials. Meta-analytic evidence confirms that although corticosteroids may deliver more effective immediate relief, platelet-rich plasma typically achieves better intermediate and long-term results, with reduced rates of repeat injections or surgical intervention [33,43].

Platelet-rich plasma (PRP) may offer advantages over corticosteroids because, in contrast to corticosteroids—which exert catabolic effects on tendon tissue and may heighten infection risk if surgical intervention follows shortly after injection—platelet-rich plasma possesses potentially anabolic properties and does not compromise tendon healing or surgical outcomes [31]. Furthermore, platelet-rich plasma demonstrates a minimal adverse event profile and can be conveniently administered in outpatient clinical settings [44]. Nevertheless, platelet-rich plasma clinical effectiveness depends on variables including leukocyte content, preparation methodology, and patient characteristics. Continued discussion exists regarding optimal platelet-rich plasma composition (leukocyte-enriched versus leukocyte-depleted), and variability in research protocols restricts definitive determinations. Importantly, platelet-rich plasma does not reliably exceed the efficacy of physical therapy, which continues to serve as a fundamental component of conservative treatment strategies.

Stem cell interventions, especially those employing mesenchymal stem cells derived from bone marrow or adipose tissue, represent an evolving regenerative strategy for chronic rotator cuff tendinopathy, though substantial clinical validation remains incomplete [35,37–40,45]. Preclinical investigations and preliminary clinical trials indicate that mesenchymal stem cells can regulate the tendon microenvironment, facilitate tissue remodeling, and augment tendon-bone interface healing through both direct cellular differentiation and paracrine immunomodulatory mechanisms [12,14,40,52]. Animal research has revealed enhanced biomechanical properties and fibrocartilage regeneration with mesenchymal stem cell-based interventions, and human investigations report improvements in pain levels, functional capacity, and tendon structural integrity following mesenchymal stem cell injections or surgical augmentation [53,54]. For instance, intratendinous administration of autologous adipose-derived mesenchymal stem cells in patients with partial-thickness rotator cuff tears produced marked pain reduction and improved tendon architecture without adverse events [54,55]. Likewise, autologous adipose-derived regenerative cell injections have demonstrated superior sustained functional outcomes compared to corticosteroids in randomized controlled trials [56].

Notwithstanding these encouraging results, clinical implementation of stem cell therapies encounters multiple obstacles. The literature reveals considerable variability in cellular sources, processing techniques, dosing regimens, and administration methods, which hinders standardization and cross-study comparison. While meta-analyses and systematic reviews suggest that mesenchymal stem cell therapies may provide the most consistent regenerative effects among orthobiologic options, including platelet-rich plasma and peptide-based approaches, substantial costs, regulatory constraints, and ethical considerations restrict broad clinical application [57]. Additionally, the American Medical Society for Sports Medicine and recent consensus evaluations stress that, despite potential for pain reduction and functional enhancement, the efficacy of stem cell therapies for tendon pathology remains undetermined due to insufficient large-scale, rigorously designed randomized controlled trials [50]. Consequently, while stem cells constitute a promising therapeutic direction for chronic rotator cuff tendinopathy management, additional investigation is necessary to establish standardized treatment protocols, long-term safety profiles, and conclusive clinical effectiveness.

Peptide-based interventions represent a developing approach in regenerative treatment of chronic rotator cuff tendinopathy, with preliminary evidence indicating potential advantages in soft tissue restoration and inflammatory regulation. Peptides, composed of short amino acid sequences, function as signaling molecules that facilitate cellular proliferation, neovascularization, and extracellular matrix restructuring. Both oral and intra-articular peptide preparations have undergone investigation, with intra-articular delivery providing targeted local effects and oral administration offering systemic advantages. Nevertheless, constraints in bioavailability and absorption pose ongoing challenges for clinical implementation. Available literature suggests that peptide therapies may represent feasible alternatives before surgical management, demonstrating encouraging results in soft tissue regeneration, though broader clinical utilization requires additional research to refine dosing strategies, delivery mechanisms, and extended safety profiles [36,40].

Preclinical and preliminary clinical investigations have examined self-assembled peptides and peptide-based scaffolds, frequently combined with other biological agents such as platelet-rich plasma, to augment tendon repair. For instance, animal research has shown that combining self-assembled peptides with platelet-rich plasma can enhance collagen structural organization, attenuate inflammation, and diminish apoptosis in rotator cuff tears, indicating a synergistic effect potentially translating to improved structural and functional results. Despite these promising observations, peptide therapies remain in early developmental stages compared to more established orthobiologic options like platelet-rich plasma and stem cells. Large-scale randomized clinical trials are essential to develop standardized treatment protocols and validate long-term efficacy and safety before peptides can be routinely advocated for chronic rotator cuff tendinopathy management [53,54,58].

## 4. Comparative Outcomes

**Figure 2:**
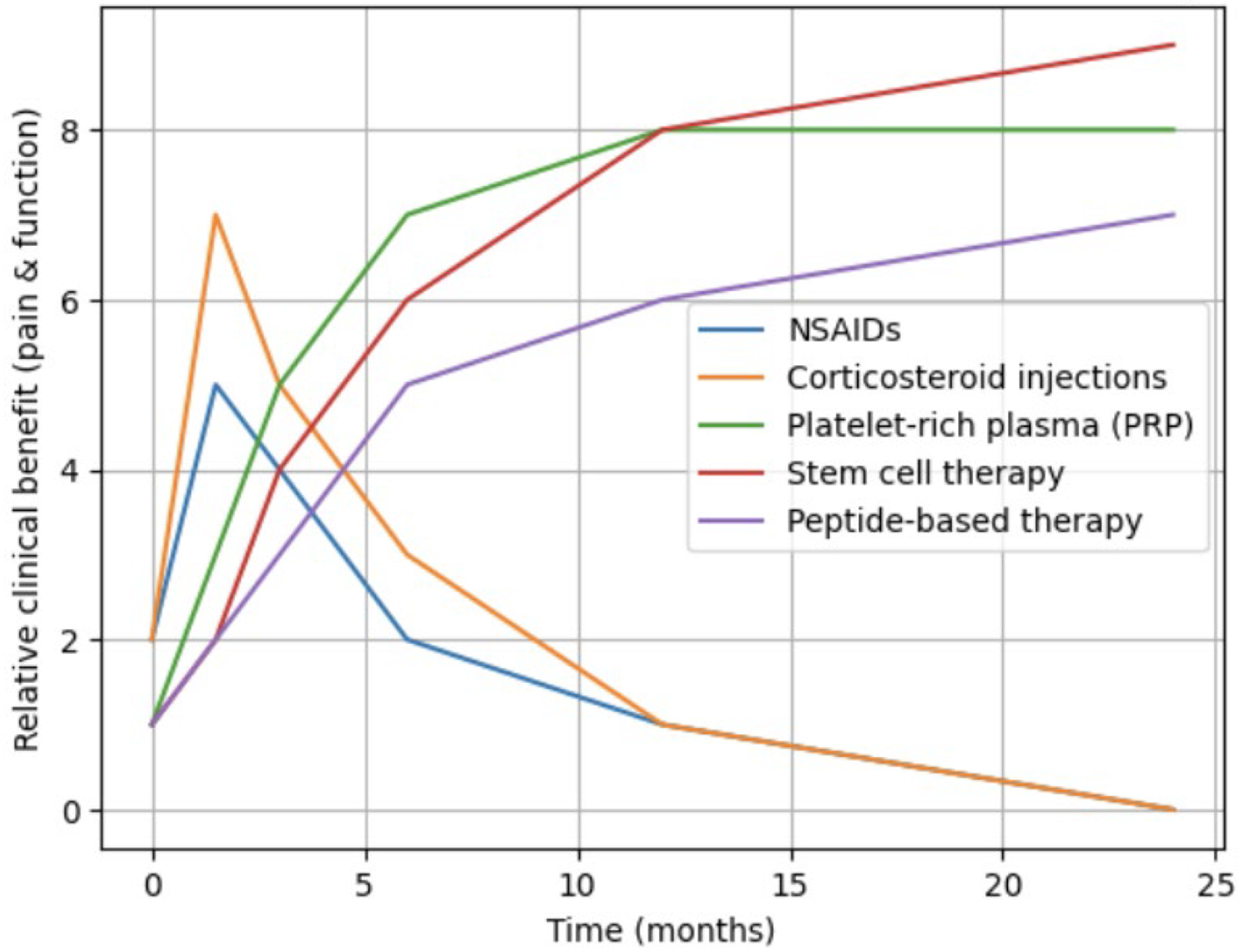
Comparative clinical benefit in regard to pain and function over time of pharmacologic versus regenerative therapies in chronic rotator cuff tendinopathy. NSAIDs, non-steroidal anti-inflammatory drugs.

Regenerative interventions such as particularly platelet-rich plasma and adipose-derived stem cells yield comparable or superior sustained outcomes in pain control, functional capacity, and structural repair relative to pharmacological treatments (especially corticosteroids) for chronic rotator cuff tendinopathy, although the clinical benefit magnitude is frequently modest and may not consistently achieve minimal clinically important thresholds [59]. Platelet-rich plasma injections typically deliver enhanced intermediate and extended pain relief compared to corticosteroids, which demonstrate greater short-term effectiveness but diminishing efficacy over time. Meta-analyses and randomized trials reveal that platelet-rich plasma-mediated pain reduction persists at 6–12 months, whereas corticosteroid benefits dissipate within several weeks. Adipose-derived stem cell therapy similarly produces durable pain amelioration at 33–40 months, surpassing corticosteroid performance [30] (Figure 2).

Regarding functional outcomes, platelet-rich plasma and stem cell therapies yield greater or equivalent functional restoration compared to corticosteroids at intermediate and long-term assessments. Platelet-rich plasma demonstrates superior improvement across validated outcome measures (UCLA, QuickDASH, Constant-Murley, ASES) at 6–12 months. Adipose-derived stem cells produce elevated ASES scores and enhanced range of motion at extended follow-up intervals [43]. For structural repair, platelet-rich plasma correlates with reduced retear rates and enhanced tendon integrity on imaging studies, indicating improved structural healing compared to pharmacological alternatives. Stem cell therapy likewise exhibits MRI evidence of tissue regeneration. Conversely, corticosteroids, despite anti-inflammatory properties, may exert catabolic effects on tendon tissue and fail to facilitate healing processes [12,16,60,61].

Regenerative interventions including platelet-rich plasma and stem cell-based therapies generally exhibit equivalent or improved safety profiles compared to pharmacological management (particularly corticosteroid injections and NSAIDs) in chronic rotator cuff tendinopathy, demonstrating fewer severe adverse events and diminished risk of tendon deterioration [62].

Multiple randomized controlled trials and meta-analyses confirm that platelet-rich plasma injections maintain safety, without significant treatment-related adverse events relative to corticosteroid injections. Being autologous, platelet-rich plasma reduces immunogenic risk and avoids the tendon catabolism or elevated infection risk associated with corticosteroids, particularly when surgery occurs within three months post-injection. Corticosteroids, despite providing short-term pain relief, carry risks of tendon weakening, potential rupture, and systemic complications including infection and impaired healing [12].

Stem cell therapies, especially autologous adipose-derived regenerative cells, likewise demonstrate excellent safety profiles with no greater risk than corticosteroid injections in available investigations. Adverse events remain rare, with no increased complication rates reported in the current literature.

Pharmacological agents such as NSAIDs present well-established systemic risks: including renal, cardiovascular, and gastrointestinal complications, particularly with extended use, while opioids are generally discouraged due to their unfavorable risk profile and absence of superior efficacy [63].

Peptide-based interventions, though less extensively studied, suggest favorable safety characteristics comparable to other regenerative modalities [33]. Overall, regenerative therapies offer a safer alternative to conventional pharmacologic management, avoiding the tissue-degenerative effects and systemic complications associated with traditional medical treatments for chronic rotator cuff tendinopathy.

Regenerative treatments, including platelet-rich plasma and stem cell-based therapies, offer safety profiles that match or exceed those of conventional pharmacologic approaches—particularly corticosteroid injections and NSAIDs—in managing chronic rotator cuff tendinopathy. These biologics produce fewer serious adverse events and pose less risk of tendon deterioration.

Evidence from numerous randomized controlled trials and meta-analyses confirms that PRP injections are well-tolerated, with no significant treatment-related complications when compared to corticosteroids [3]. Because PRP is derived from the patient’s own blood, it carries minimal immunogenic risk and avoids the catabolic effects on tendon tissue associated with steroids. Corticosteroids, while providing short-term pain relief, are linked to tendon weakening, potential rupture, increased infection rates (particularly if surgery occurs within three months post-injection), and various systemic side effects including impaired healing [16,29,30].

Stem cell interventions, especially those using autologous adipose-derived regenerative cells, demonstrate similarly strong safety outcomes, with complication rates no higher than corticosteroid injections and very few reported adverse events [44,63].

In contrast, pharmacologic options carry well-documented systemic risks. NSAIDs, particularly with chronic use, are associated with renal, cardiovascular, and gastrointestinal toxicity. Opioids are not recommended due to their unfavorable risk-benefit ratio and lack of superior therapeutic effect [44]. Peptide-based therapies, though less extensively studied, appear to share the favorable safety characteristics of other regenerative modalities [42].

## 5. Practical Considerations

### 5.1 Practical Limitations of Stem Cell and Peptide-Based Therapies

Despite promising clinical outcomes, stem cell and peptide-based interventions for chronic rotator cuff tendinopathy face significant translational barriers that currently limit their widespread clinical implementation. These obstacles—encompassing economic, logistical, and regulatory dimensions—contrast markedly with the relative accessibility of platelet-rich plasma and conventional pharmacologic treatments.

The economic burden of stem cell therapies represents a primary constraint to adoption. Mesenchymal stem cell interventions typically cost several thousand dollars per treatment due to complex requirements for cell harvesting, laboratory processing, quality control, and specialized delivery systems.These expenses are compounded by minimal insurance reimbursement, effectively restricting access to patients with substantial out-of-pocket resources [64]. Peptide-based therapies present similar financial challenges, driven by proprietary synthesis methods and limited commercial production [65].

Accessibility remains equally problematic. While PRP has achieved broad integration into routine orthopedic practice through relatively simple, standardized preparation protocols, stem cell therapies require sophisticated laboratory infrastructure, specialized technical expertise, and strict quality assurance mechanisms. Consequently, these interventions remain predominantly confined to tertiary care centers and academic research settings [51,55]. Peptide-based treatments are in even earlier stages of clinical translation, with availability largely restricted to investigational protocols and select private practices [66].

Regulatory frameworks present additional substantial hurdles. The FDA classifies most stem cell products as biologics requiring extensive preclinical and clinical validation prior to approval for routine clinical use. This classification mandates rigorous Phase I-III trials, effectively limiting current stem cell applications to investigational settings with appropriate regulatory oversight [52]. Peptide-based interventions face comparable regulatory scrutiny, with few products currently authorized for musculoskeletal indications. By contrast, PRP—as an autologous, minimally manipulated blood product—operates under less stringent regulatory requirements under current FDA guidance, facilitating more rapid clinical adoption [32].

These multifactorial barriers collectively impede the translation of stem cell and peptide-based therapies from promising experimental interventions to accessible clinical treatments, underscoring the need for continued research, standardization efforts, and policy evolution to realize their therapeutic potential.

### 5.2 Patient Selection

The literature demonstrates consistent patient selection criteria across regenerative therapy studies for chronic rotator cuff tendinopathy, with eligible candidates typically presenting with persistent symptoms despite at least three months of structured conservative care, partial-thickness tears confirmed by imaging, and documented failure of nonoperative management [67–69]. This temporal threshold distinguishes acute from chronic pathology suitable for regenerative approaches, ensuring that less invasive options including physical therapy, NSAIDs, and activity modification have been exhausted before advancing to biologic interventions [60,67].

MRI or ultrasound-confirmed partial-thickness rotator cuff tears and tendinopathy without complete disruption represent standard inclusion criteria across most investigations, while full-thickness tears with retraction, massive tears, and advanced degeneration are typically excluded due to concerns about biological healing capacity and mechanical limitations that may compromise regenerative potential [68]. PRP studies predominantly focus on chronic tendinopathy and partial tears unresponsive to physical therapy, systematically excluding complete or retracted tears [69]. Stem cell investigations, particularly those using bone marrow concentrate, employ similar criteria, targeting non-retracted partial or small full-thickness tears refractory to exercise therapy [70]. Evidence for peptide-based interventions remains limited, though emerging studies generally mirror established selection frameworks for PRP and cellular therapies, reflecting a consistent approach to patient eligibility across regenerative modalities.

## 6. Conclusion

Current evidence suggests that regenerative therapies, particularly mesenchymal stem cell and platelet-rich plasma interventions, show promise for chronic rotator cuff tendinopathy, with MSC therapies demonstrating the most consistent regenerative effects including significant pain reduction and preliminary evidence of enhanced tendon healing and reduced retear rates in both preclinical and early clinical studies, while PRP interventions yield moderate, sustained improvements in pain and function compared to corticosteroids, particularly at long-term follow-up [29,31,49]. Peptide-based therapies represent emerging modalities with early promising data but sparse clinical evidence [1]. In contrast, conventional pharmacologic management—including corticosteroid injections and NSAIDs—provides only short-term symptomatic relief without addressing underlying tendon pathology, with corticosteroids demonstrating no long-term superiority over physical therapy and potential adverse effects on tendon integrity [71,72]. However, clinical adoption of regenerative therapies remains constrained by critical limitations including absence of standardized protocols, marked heterogeneity in cell sources, preparation methods, dosing regimens, and outcome measures, and insufficient high-quality randomized controlled trials [50,51]. There is clear consensus that large-scale, methodologically rigorous comparative studies are essential to establish efficacy, optimize safety profiles, and refine patient selection criteria, with future research directions including personalized treatment strategies incorporating immune profiling and advanced biomaterial delivery systems to enhance regenerative outcomes [61].

Chronic rotator cuff tendinopathy is a degenerative condition characterized by pain, weakness, and impaired shoulder function, in which traditional pharmacologic treatments such as NSAIDs and corticosteroid injections offer only short-term relief without promoting tendon healing—and may even worsen tendon integrity, particularly with repeated steroid use. These limitations have driven interest in regenerative options like platelet-rich plasma, stem cells, and peptide-based therapies, which show greater potential for long-term improvement in pain, function, and tendon structure. PRP consistently provides superior intermediate and long-term outcomes compared to steroids, while stem cell therapies demonstrate promising but still early evidence of enhanced tissue repair, though both face cost, regulatory, and accessibility barriers. Peptide therapies are emerging but require substantial further validation. Overall, regenerative approaches appear safer and more durable than conventional pharmacologic management, especially for patients with chronic symptoms and partial-thickness tears who have failed conservative care.

## 7. Key Points

- Chronic rotator cuff tendinopathy is primarily a degenerative, rather than inflammatory, condition.
- NSAIDs and corticosteroid injections provide short-term symptom relief but do not promote tendon healing.
- Repeated corticosteroid injections may compromise tendon integrity and worsen long-term outcomes.
- Platelet-rich plasma demonstrates superior intermediate- and long-term pain and functional outcomes compared to corticosteroids.
- PRP has a favorable safety profile and does not impair tendon healing or surgical outcomes.
- Mesenchymal stem cell therapies show promising regenerative and structural benefits but lack standardized protocols.
- High cost, limited insurance coverage, and regulatory barriers restrict widespread stem cell use.
- Peptide-based therapies are emerging regenerative options with early supportive data but insufficient clinical validation.
- Regenerative therapies may reduce the need for repeat injections or surgical intervention in select patients.
- Large-scale, high-quality randomized controlled trials are needed to establish optimal protocols and long-term efficacy.
